# Multiterminal En Plaque Motor Endplates in Extraocular Muscles Are Conserved Across Vertebrate Species

**DOI:** 10.1167/iovs.66.4.77

**Published:** 2025-04-28

**Authors:** Jing-Xia Liu, Abraha Kahsay, Nils Dennhag, Jonas von Hofsten, Fatima Pedrosa Domellöf

**Affiliations:** 1 Department of Medical and Translational Biology, Umeå University, Umeå, Sweden; 2 Department of Clinical Sciences, Ophthalmology, Umeå University, Umeå, Sweden

**Keywords:** extraocular muscle, zebrafish, rabbit, mice, motor endplate

## Abstract

**Purpose:**

We have previously described a novel type of multiterminal en plaque motor endplates in the human extraocular muscles (EOMs). This study aimed to investigate whether multiterminal en plaque motor endplates are conserved in EOMs among vertebrates.

**Methods:**

The motor endplates were identified with α-bungarotoxin (α-BTx) and antibodies against synaptic proteins and neurofilament in the EOMs of zebrafish, rabbits and mice. Transcriptomic data were re-analyzed to identify acetylcholine receptor (AChR) subunits in EOMs and trunk muscles of wild-type zebrafish at five and 20 months of age.

**Results:**

In addition to the two typical types of single en plaque and multiple en grappe motor endplates, the third type of multiterminal en plaque motor endplates were observed in the EOMs of zebrafish, rabbits, and mice. The EOMs of zebrafish showed a significantly higher proportion of myofibers containing multiterminal en plaque motor endplates compared to EOMs of rabbits and mice. RNA sequencing data revealed significantly higher AChR subunits in the zebrafish EOMs compared to trunk muscles.

**Conclusions:**

Multiterminal en plaque motor endplates are not exclusive to human EOMs but are also present in the EOMs of other vertebrate species, suggesting a conserved feature of the EOMs.

The extraocular muscles (EOMs) are responsible for a variety of eye movements. They maintain visual alignment and possess distinct physiological and anatomical characteristics that set them apart from trunk and limb skeletal muscles.^1^^,^^2^ The physiological uniqueness of EOMs is reflected in their higher innervation density and very small size of motor units, along with extremely fast contractile properties, large variability in myofiber types, and fatigue resistance.^3^^–^^5^ Typically, the EOMs have been considered to have two major types of motor endplates: single en plaque and multiple en grappe.^6^^,^^7^ The single en plaque motor endplates have been found in all EOM myofibers, whereas small multiple en grappe endplates are only found in myofibers containing slow-tonic myosin heavy chain (MyHC).^6^^,^^7^ These two groups of myofibers have been referred to as singly innervated myofibers (SIFs) and multiply innervated myofibers (MIFs), respectively. Unlike the other skeletal muscles of the body, which generally express only the adult acetylcholine receptor (AChR) subunit and typically only have SIFs, EOMs express both the fetal gamma and the adult epsilon AChR subunits.^4^^,^^8^^,^^9^ The composition of the EOMs regarding MyHC isoforms, the major contractile proteins, is far more complex than that of the other skeletal muscles.^5^^,^^10^^,^^11^ In adult human EOMs, although a variety of MyHC isoforms can be present in one single myofiber,^5^^,^^11^^,^^12^ three major myofiber types can be identified based on the content of MyHC isoforms (i.e., slow myofibers containing MyHCI and MyHCslow tonic [MyHCsto/I], fast myofibers containing MyHCIIa, and myofibers lacking these isoforms but containing MyHC extraocular [MyHCeom]).^9^^,^^13^ In zebrafish, EOMs display a majority of fast myofibers containing fast MyHC, slow myofibers containing slow MyHC isoform, followed by hybrid myofibers containing both fast and slow MyHC isoforms, and EOM-like myofibers lacking fast and slow MyHCs.^14^

We have reported a novel type of motor endplates in human EOMs: the multiterminal en plaque motor endplates, in both the global and orbital layers, which are both abundant and specifically present in myofibers containing MyHCeom.^9^ In contrast to the small size of multiple en grappe motor endplates, the multiterminal en plaque motor endplates are larger, with a greater distance between adjacent endplates. The length of each motor endplate ranges from approximately 15 to 40 µm, and the distance between adjacent endplates varies from approximately 25 to 240 µm. Additionally, some of the multiterminal en plaque motor endplates are located on opposite sides of the longitudinally sectioned muscle fibers.^9^

The presence of multiterminal en plaque innervation has, to our knowledge, only been described thoroughly in human EOMs.^9^ The present study aimed to determine whether multiterminal en plaque motor endplates are present in EOMs of other vertebrate species commonly used to study the EOMs.

## Material and Methods

### Muscle Samples

The study was carried out according to national and international guidelines and complied with the ARRIVE guidelines for the use of animals in research. Zebrafish (Danio rerio), rabbit, and mouse experiments conformed to the ARVO Statement for the use of Animals in Ophthalmic and Vision Research and were performed with approval of the Animal Review Board at the court of Appeal of Northern Norrland in Umeå (zebrafish: Dnr: A6 2020; rabbit: Dnr: A27-13; mouse: Dnr: A22-2023).

Zebrafish were maintained at the Umeå University Zebrafish Facility. Twenty-eight month-old wild type and transgenic lines *Tg(mylz2:GFP)^i135^*, labeling fast myofibers and *Tg(smyhc1:tdTomato)^i261^*, labeling slow myofibers, were anaesthetized using tricaine until they were unresponsive to touch but retained a strong heartbeat. Immediately afterward, both eyes from each fish were carefully excised using microdissection surgical scissors and fixed in 4% paraformaldehyde to be processed for subsequent whole-mount immunostaining. In total, 24 eyes from 12 fish were collected and analyzed.

Following RRR (Reduce, Replace, Refine) principles, the mammals used were rabbits and mice that served as wild-type (WT) controls, in whom no experiments whatsoever were performed. Five adult New Zealand white rabbits (weight 2.5–3.1 kg) were euthanized by intraperitoneal injection of 200 mg/kg of pentobarbital, and six EOM muscles were obtained. Four mice (∼28 days old) with mixed WT background (129/Sv:CBA/J:C57BL/6 J:DBA2/J)^15^ were killed by cervical dislocation, and eight EOM samples were collected. The muscle specimens from both mammal groups were oriented and directly mounted on thin cardboard with OCT compound (Tissue Tek; Miles Laboratories, Naperville, IL, USA), rapidly frozen in propane chilled with liquid nitrogen, and stored at −80°C until use. Care was taken to orientate the EOM specimens to allow the longitudinal sections to contain both the orbital and global layers. Serial longitudinal sections were cut with a cryostat microtome (Reichert-Jung; Leica, Heidelberg, Germany) at −23°C. The sections were 40 µm thick for confocal microscopy and 7 µm thick for fluorescence light microscopy.

### Antibodies and Immunofluorescence

Primary and secondary antibodies used in the present study are summarized in Supplementary Table S1. Motor endplates were identified by labeling with α-bungarotoxin (α-BTx), which binds to post-synaptic AChRs on the plasma membrane of myofibers and/or with the mixtures of antibodies against pre-synaptical proteins (synaptic vesicles or synaptophysin) and neurofilaments which identify the axonal side of neuromuscular junctions (NMJs). Using a mixture of antibodies against synaptical proteins and neurofilament allowed the simultaneous labeling of motor endplates and the axon at NMJs using a single fluorochrome. Specifically, Alexa Fluor 647-conjugated (B35450) or tetramethylrhodamine-conjugated α-BTx (T1175; Invitrogen, Molecular Probes Inc., Eugene, OR, USA) were used to identify motor endplates in EOMs of all three species. In zebrafish EOMs, monoclonal antibody SV2 against synaptic vesicle glycoprotein 2A (Developmental Studies Hybridoma Bank, Iowa City, IA, USA) or a mixture of SV2 and mouse monoclonal anti-acetyl-alpha tubulin (α-tubulin) antibody recognizing axonal nerve fibers (T7451; Sigma-Aldrich, St. Louis, MO, USA) were used to label the presynaptic region of the NMJs. A mixture of mouse monoclonal antibodies SY38 against synaptophysin (Boehringer Mannheim, Indianapolis, IN, USA) and M0762 against neurofilament 70 kDa (Dako Denmark A/S, Glostrup, Denmark) were used to detect NMJs by labeling the pre-synaptic region in EOMs of rabbits and mice.

Zebrafish transgenic lines *Tg(mylz2:GFP)^i135^ and Tg(smyhc1:tdTomato)^i261^* were used to visualize and classify myofiber types. The former contained green fluorescent protein (GFP) specifically in fast myofibers, and the latter expressed tdTomato bright red fluorescent protein in slow myofibers.^14^ In wild type zebrafish, all myofibers were visualized by labeling with Alexa Fluor 488 Phalloidin (A12379; Fisher Scientific, Gothenburg, Sweden) and mouse monoclonal antibody S58 (Developmental Studies Hybridoma Bank) recognizing slow myofibers was also used. Obscurin IQ (gift of Prof. Matias Gautel, King's Collage, London, UK), a rabbit polyclonal antibody was used to label myofibers in mouse EOM and chicken polyclonal antibody against laminin (LS-C96142; LSBio, Shirley, MA, USA) was used to visualize the contour of rabbit EOM myofibers.

Zebrafish immunostaining was performed as previously described.^16^ Whole-mount EOMs were washed in PBS with 0.1% Tween 20 and then pre-incubated in blocking buffer containing 1% blocking reagent (Roche Diagnostics GmBH, Manheim, Germany), 1% dimethyl sulfoxide and 5% sheep serum in PBS with 0.4% TritonX for at least one hour at room temperature. The pre-blocking solution was removed and replaced with primary antibodies for incubation at 4°C overnight. After phosphate buffer saline with tween 20 (PBT) washing, whole-mount EOMs were incubated with secondary antibodies or directly conjugated high-affinity ligands at 4°C overnight (Supplementary Table S1). Unbound secondary antibody was washed out with PBT, and EOMs were cleared and mounted in 70% glycerol (Sigma-Aldrich).

Double- or triple-immunolabelings were performed on air-dried longitudinal tissue sections in EOMs of rabbits and mice, as previously described.^16^ In brief, tissue sections were rehydrated in 0.01M PBS and then blocked with 5% donkey normal serum for 15 minutes. Sections were then incubated with the appropriate primary antibody at 4°C overnight or for 60 minutes at 37°C. All antibodies were diluted in 0.01M PBS containing 0.1% bovine serum albumin and used at their optimal dilutions (Supplementary Table S1). After washing in PBS and an additional blocking with 5% donkey normal serum for 15 minutes, sections were incubated for one hour at 37°C with the appropriate secondary antibody and finally washed in PBS and covered with Vectashield mounting medium (Vector Laboratories, Inc., Burlingame, CA, USA). The secondary antibodies used were conjugated with Alexa Fluor 488, Alexa Fluor 647 (Molecular Probes, Inc., Eugene, OR, USA) or Rhodamine Red-X (Jackson ImmunoResearch Europe Ltd., Newmarket, UK). Control sections were treated as above, except that the primary antibody was excluded. No staining was observed in the control sections.

### Re-Analysis of RNA-Sequencing Data

For details on EOM and trunk muscle isolation, RNA extraction and sequencing, we refer to the original publication.^15^ To investigate AChR transcript levels, we excluded all *desmin* KO samples from the original data set and re-analyzed data using only WT samples. In total, 12 samples were used including five-month-old EOMs and trunk muscles and 20-month-old EOMs and trunk muscles. DEseq2^17^ was used to perform differential expression analysis, adjusted *P*-value of 0.01 was considered significant. Statistical analysis was performed using the original DEseq2 settings, using a Benjamini-Hochberg corrected Wald test for multiple testing.

### Confocal and Light Microscopy

Whole-mount EOMs of zebrafish and thick (40 µm) longitudinal muscle sections of mice or rabbit EOMs were examined and photographed using a Nikon A1 confocal microscope (Nikon, Tokyo, Japan). The thin (7 µm) muscle sections from rabbit and mouse EOMs were examined under a Leica DM 6000 B microscope (Leica Microsystems, Wetzlar, Germany). Images were processed using Fiji ImageJ (https://imagej.net/software/fiji/)^18^ and Adobe Photoshop CS6 software (Adobe Systems, San Jose, CA, USA).

### Statistical Analysis

Quantification of NMJs and myofibers of the EOMs and the measurement of NMJ length was done using Fiji ImageJ (https://imagej.net/software/fiji/).^18^ Data were collected in Microsoft Excel, and plotted in GraphPad Prism 10 and RStudio. Statistical analysis was conducted using one-way ANOVA followed by post hoc *t*-test, *P* ≤ 0.05 was considered significant (*P ≤ 0.05, **P ≤ 0.01, ***P ≤ 0.001, ****P ≤ 0.0001). Data are presented as mean ± SEM.

## Results

The EOM lengths examined varied among zebrafish, rabbits and mice. All motor endplates were indentified along almost the entire length of the whole-mount EOMs (approximately 1918 µm), extending from the posterior to the anterior regions. However, in contrast to zebrafish, EOMs were examined using incomplete muscle samples in rabbits and mice. Thus the maxium length of a single myofiber examined from the available piece of muscle samples was approximately 13,000 µm in rabbits, and up to 1878 µm in mice, which did not include the ends of the EOMs. Therefore it is worth noting that the mouse EOM samples used in this study were shorter than those from zebrafish, because the mouse EOM samples did not cover the full myofiber length of the muscles from the origin to the insertion on the eye. Therefore the numbers of myofibers with typical small en grappe motor neurons may be considerably underestimated. This also applies to the rabbit samples, although to a lesser degree. This methodological limitation could potentially affect the overall analysis of NMJ distribution along the entire muscle length. However, we clearly identified the three different innervation patterns in the examined animals. A relation to myofiber type and motor endplate type was not possible to establish considering the limitation inherent to the number of antibodies needed and their species specificities.

### Zebrafish

There are some differences in the organization of the EOMs between zebrafish and mammals.^14^ For example, the orbital and global layers are not clearly defined in zebrafish EOMs, whereas the two layers are well defined in EOMs of humans, rabbits, and mice. However, the two layers in zebrafish EOMs can be distinguished based on the size of myofibers: the global layer contains large fast myofibers, whereas orbital layer contains small fast, as well as slow, myofibers. In addition, the slow myofibers are present only in the orbital layer, as described in our previous studies.^14^

**Figure 1.**
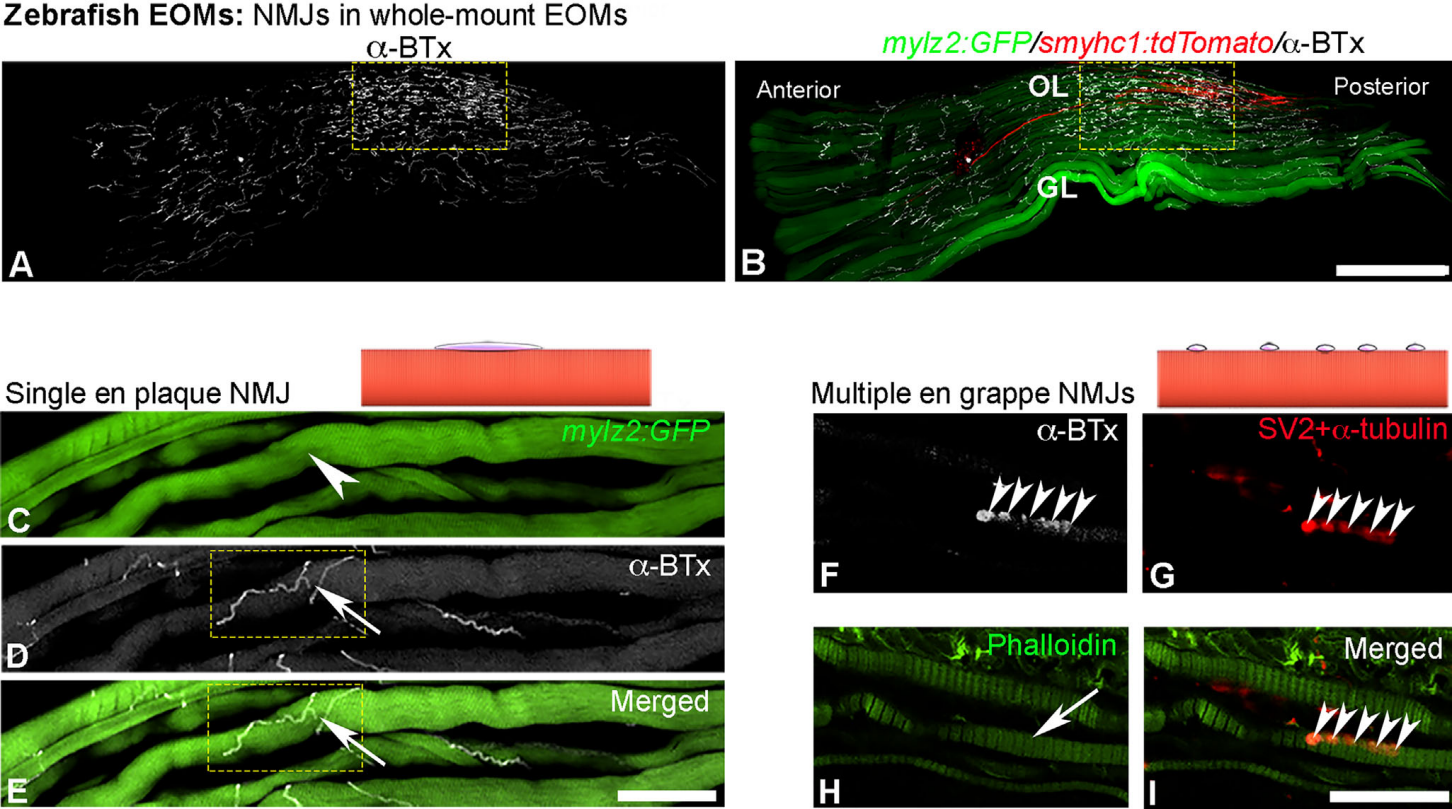
Representative confocal images and schematic illustrations showing single en plaque or multiple en grappe motor endplates in whole-mount EOMs from zebrafish. (**A**, **B**) Longitudinal view of whole-mount EOM taken with confocal microscope from the top, where large fast myofibers dominate the global side of EOMs (GL), whereas small fast and slow myofibers are restricted to the orbital layer (OL). NMJs labeled with α-BTx (*white* in **A** and **B**) in double transgenic lines, *Tg(mylz2:*GFP), *green* in **B**, identifies all fast myofibers in both GL and OL and *Tg(smyhc1:*tdTomato), *red* in **B**, identifies all slow myofibers. Note the enrichment of NMJs in OL close to the nerve entrance (framed area in **A** and **B**). (**C**–**E**) Single en plaque motor endplate (*arrows* in **D** and **E** within the framed area) labeled with α-BTx (*white* in **D** and **E** within the framed area) on a fast myofiber (*arrowhead* in **C**), depicted also by schematic illustration. (**F**–**I**) Multiple en grappe motor endplates labeled with α-BTx (*white* in **F** and mixture of SV2 with α-tubulin (*red* in **G**) on a myofiber identified with phalloidin labeling (*arrow* in **H**), depicted also by schematic illustration. *Scale bars*: 300 µm in **B**, 100 µm in **E**, and 25 µm in **I**.

The NMJs were distributed along the entire length of the whole-mount EOMs (Figs. 1^2^–3), extending from the posterior to the anterior regions. Notably, a high number of NMJs was observed close to the nerve entrance to the muscle (Figs. 1A–B) in the OL, where slow and small fast myofibers were most abundant.

**Figure 2.**
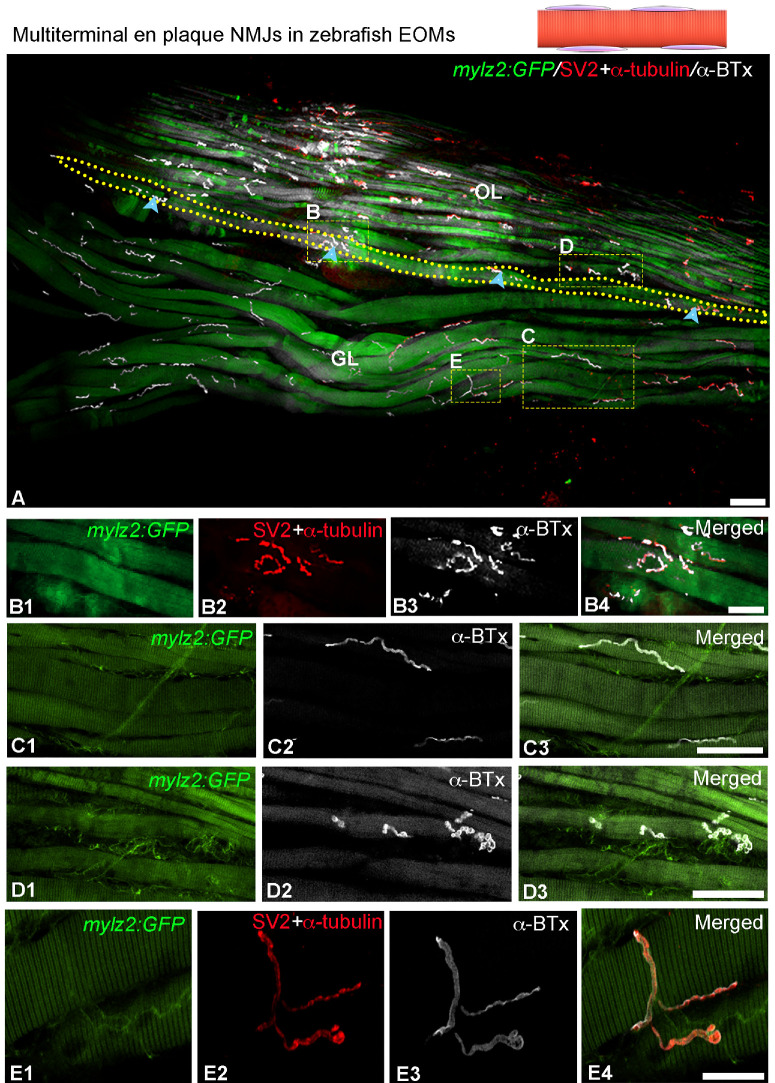
Confocal images and schematic illustration showing the distribution of multiterminal en plaque motor endplates in whole-mount EOMs. NMJs were identified with α-BTx (in *white*) and/or a mixture of SV2 and α-tubulin (in *red*) in transgenic lines, *Tg(mylz2:*GFP), *green* in **B**, in which all fast myofibers in GL and OL are green. (**A**) A myofiber outlined by the *yellow dotted line* containing four en plaque motor endplates along its length is depicted (*blue arrowheads* in **A**). (**B**–**E**) Images with higher magnification from A show four patterns of NMJs belonging to multiterminal en plaque motor endplates: (**B1**–**B4**) typical lobulated en plaque motor endplates; (**C1**–**C3**) long linear motor endplates; (**D1**–**D3**) shorter endplates displayed on small fast myofiber in the OL; and (**E1**–**E4**) endplate branching from one myofiber to another myofiber. *Scale bar*: 50 µm in **A**, **C3**, and **D3**; 25 µm in **B4** and **E4**.

**Figure 3.**
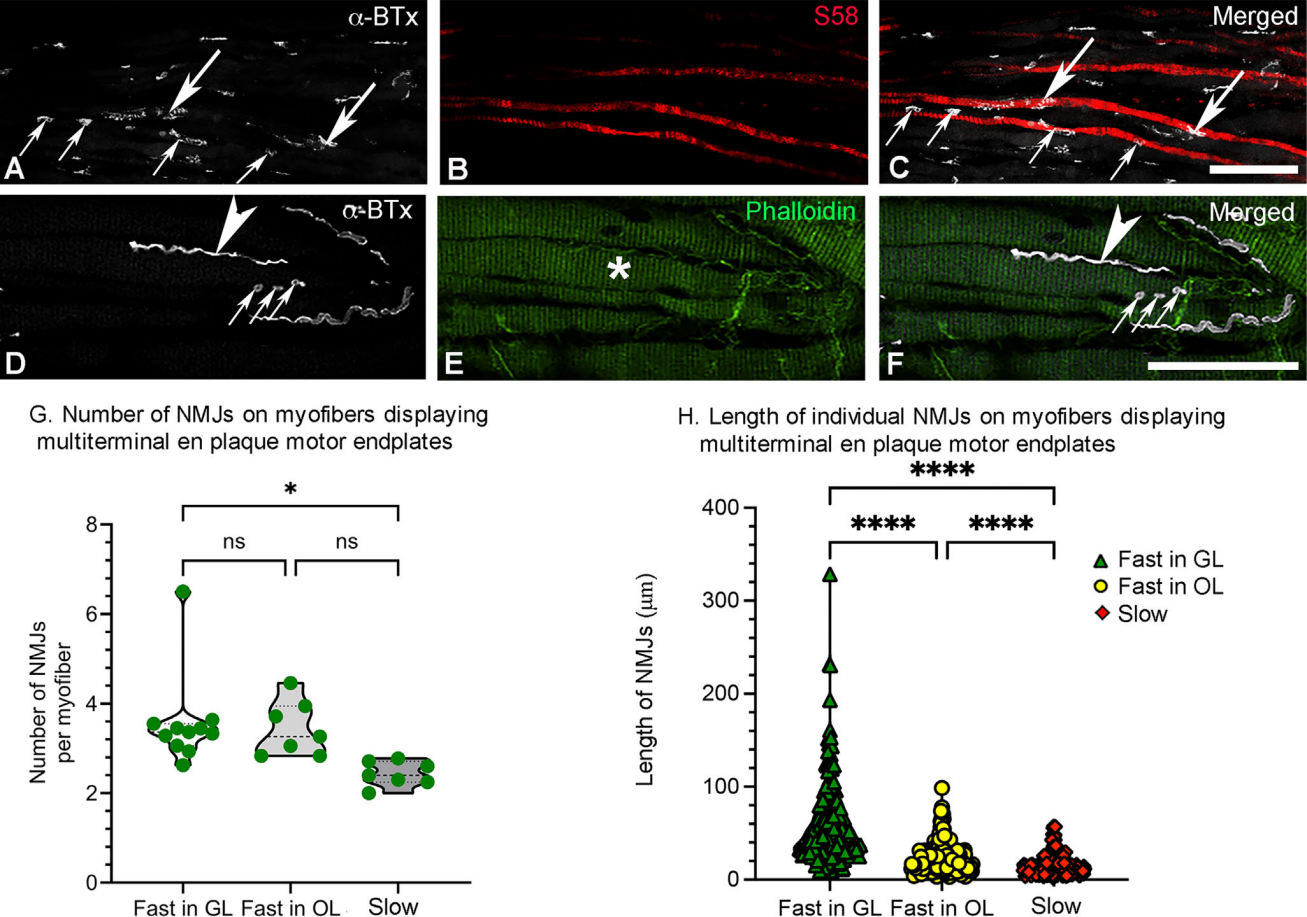
Confocal images and quantification of the number and length of individual NMJs on myofibers displaying multiterminal en plaque motor endplates in whole-mount zebrafish EOMs. (**A**–**C**) Two slow myofibers labeled with S58 (*red* in **B** and **C**) containing two and four en plaque motor endplates (*long* and *short arrows* in **A** and **C**), classified therefore as myofibers containing multiterminal en plaque motor endplates, were identified with α-BTx (*white* in **A** and **C**). (**D**–**F**) En grappe motor endplates consisting of three small NMJs labeled with α-BTx (*arrows*, *white* in **D** and **F**) and en plaque motor endplate (*arrowhead*, *white* in **D** and **F**) on an individual myofiber, which is labeled with phalloidin (*green* in **E**, marked with *asterisk*). *Scale bar*: 50 µm. (**G**) The number of NMJs on individual myofibers which are fast myofibers in both GL and OL, and in slow myofibers displaying multiterminal en plaque motor endplates. The number of zebrafish EOMs studied is given as individual symbols in each violin. (**H**) The length of individual NMJs on myofibers displaying multiterminal en plaque motor endplates, with n representing the number of myofibers examined. The average length of fast myofibers is 58.5 ± 2.8 µm in the GL, 23.1 ± 1.0 in the OL, and the average length of slow myofibers is 15.3 ± 0.8. *P ≤ 0.05; ****P ≤ 0.0001; ns: not significant.

Upon careful investigation, it was found that approximately 0.79% ± 0.4% of 430 myofibers containing NMJs, were innervated by single en plaque motor endplates (Figs. 1C–E) and 0.59% ± 0.6% myofibers were innervated by multiple en grappe motor endplates (Figs. 1F–I). The vast majority of myofibers studied (98.6% ± 0.7%) exhibited two or more en plaque motor endplates along their length, regardless of fiber type (fast or slow) or location (OL and GL) (Figs. 2, 3). Thus they were classified as multiterminal en plaque motor endplates, as reported in human EOMs.^9^

Variations in the innervating patterns of the multiterminal en plaque motor endplates, in addition to the usual pretzel-like shape (Figs. 2B1–B4), were observed across different myofiber types and layers of EOMs. Most of the motor endplates on large fast myofibers in the GL displayed linear or jagged shapes along the sarcolemma (Figs. 2C1–C3). In contrast, motor endplates on small diameter fast myofibers tended to be shorter (Figs. 2D1–D3). In addition, certain multiterminal en plaque motor endplates were ramified, with branching structures that extended along the sarcolemma of the myofiber or crossed to adjacent myofibers (Figs. 2E1–E4). The endplates on slow myofibers also tended to be shorter (Figs. 3A–C).

Both small multiple en grappe and multiterminal en plaque endplates along the length of an individual myofiber were sporadically found. This was noted in both slow and fast myofibers in both GL and OL (Figs. 3D–F), similar to the pattern reported in human EOMs previously.^9^

The distances between adjacent motor endplates ranged from 13.5 to 571 µm, and the maximum distance (571 µm) between endplates on the same myofiber was observed on a myofiber with a total fiber length of 939 µm, showing that these endplates were clearly separated from each other on a single myofiber. The number of motor endplates forming multiterminal en plaque motor endplates varied among EOM specimens, layers and myofiber types. Specifically, the number of NMJs displayed by large fast myofibers in the GL was statistically significantly higher than that of slow myofibers which were mainly localized in OL (*P* = 0.02; Fig. 3G). The number of NMJs displayed by small fast myofibers in the OL was higher than that of slow myofibers but no statistically significant difference was found (*P* = 0.06; Fig. 3G). In addition, the length of individual NMJs forming multiterminal en plaque motor endplates varied from 2.1 to 328.4 µm, with an average length of 58.5 ± 2.8 µm in fast myofibers in the GL, 23.1 ± 1.0 in fast myofibers in the OL, and 15.3 ± 0.8 in small slow myofibers (Fig. 3H). Statistical analysis showed a significant difference in the length of NMJs between fast myofibers in GL and OL (*P* ≤ 0.0001), between fast myofibers in the GL and slow myofibers (*P* ≤ 0.0001) and between fast myofibers in OL and slow myofibers (*P* ≤ 0.0001; Fig. 3H).

**Figure 4.**
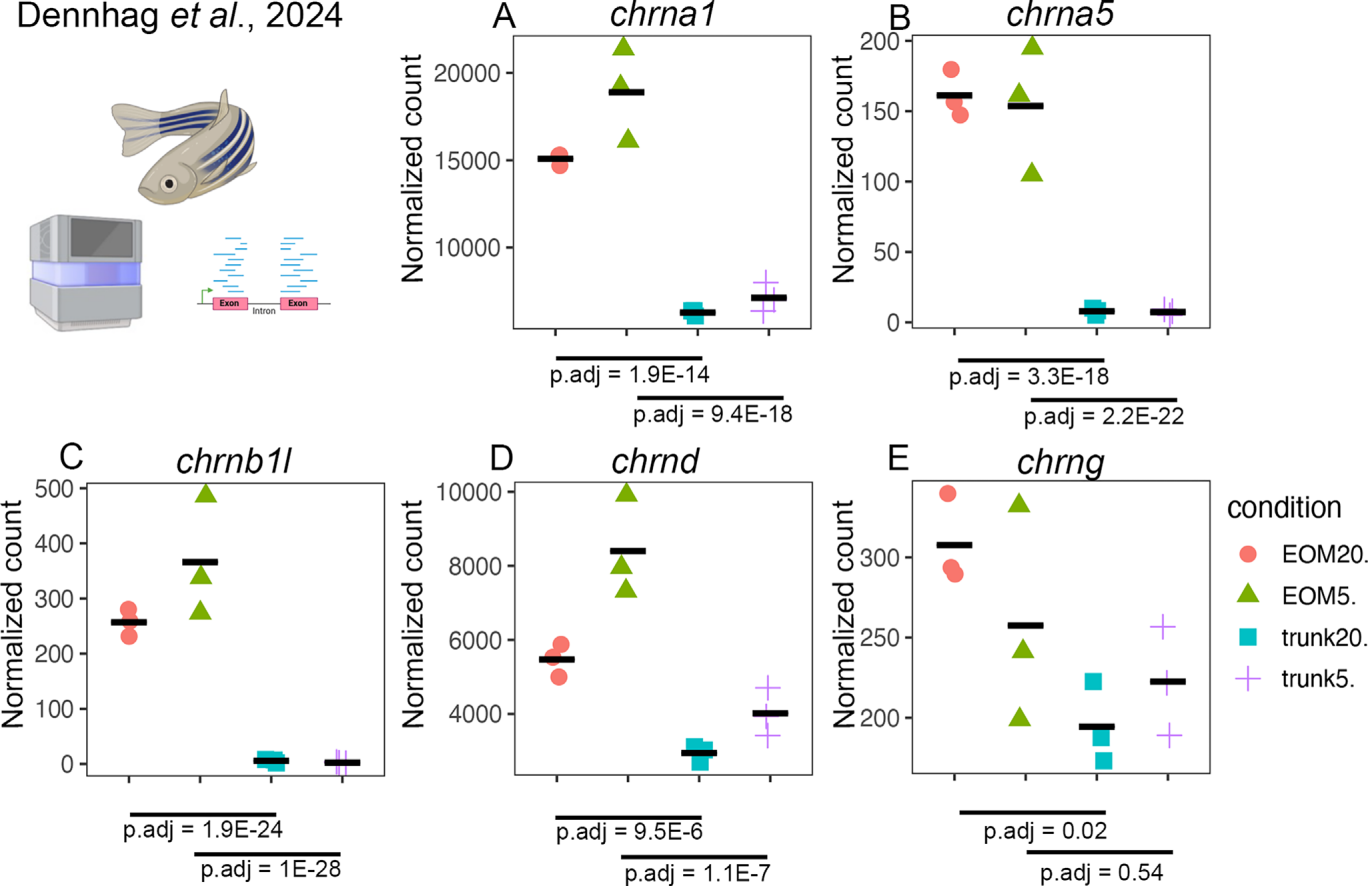
Re-analysis of a previously published RNA-sequencing data set revealed significantly higher levels of (**A**) cholinergic receptor nicotinic alpha 1 (*chrna1*), (**B**) cholinergic receptor nicotinic alpha 5 (*chrna5)*, (**C**) cholinergic receptor nicotinic beta 1 like (*chrnb1l)*, (**D**) cholinergic receptor nicotinic delta (*chrnd*), and (**E**) cholinergic receptor nicotinic gamma (*chrng)* genes in zebrafish EOMs compared to trunk muscle tissue, at five months (EOM5, trunk5) and 20 months (EOM20, trunk20).

Previous reports from us and others have shown differences in AChR subunits in EOMs compared to limb muscle tissue.^4^^,^^8^^,^^9^ To investigate whether the same differences are present in the zebrafish EOMs, we re-analyzed a recently published RNA-sequencing data set.^15^ Principal component analysis using only WT samples of the EOMs and of trunk muscles at two different ages, five and 20 months, revealed that each sample group clustered together, with the largest variance on PCA1 representing the two different tissues (Supplementary Fig. S1A), as expected. We then plotted all 19 AChR genes found across the samples to study their level of expression (Supplementary Fig. S1). We found that RNA transcripts from five subunits including cholinergic receptor nicotinic alpha 1 (*chrna1*)*,* cholinergic receptor nicotinic alpha 5 (*chrna5*)*,* cholinergic receptor nicotinic beta 1 like (*chrnb1l*)*,* cholinergic receptor nicotinic delta (*chrnd*), and cholinergic receptor nicotinic gamma (*chrng*, corrresponding to the only fetal isoform) were specifically more abundant in the EOMs compared to trunk muscles (Figs. 4A–E), whereas only one subunit, cholinergic receptor nicotinic beta 2 (*chrnb2*), was significantly more abundant in trunk muscles over both time points (Supplementary Fig. S1M). The other α-, β-subunits and cholinergic receptor nicotinic epsilon (*chrne*) did not show significant differences between EOMs or trunk muscles (Supplementary Fig. S1), consistent with results from human EOMs.^9^

### Rabbit

**Figure 5.**
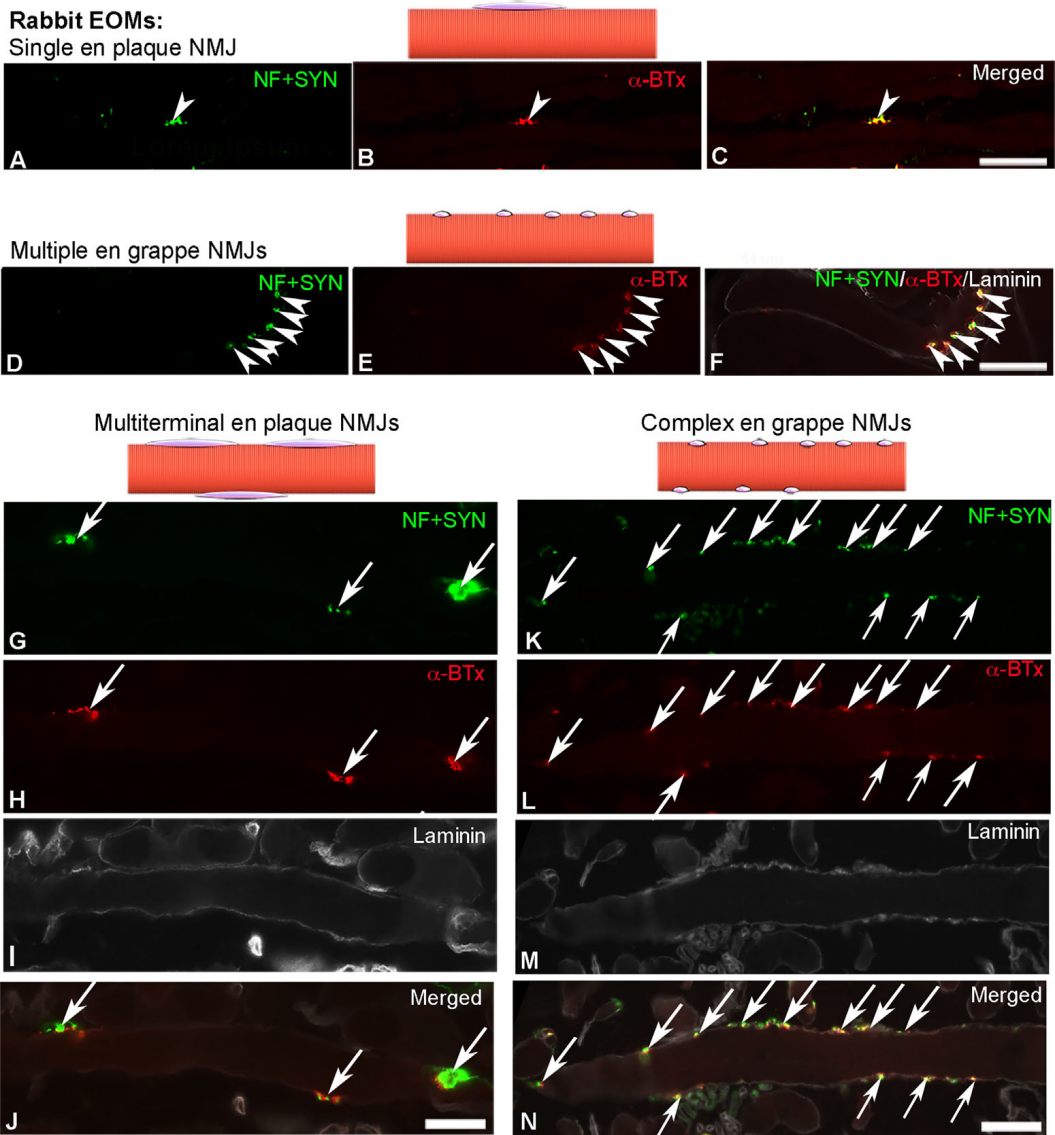
Light microscopy images and schematic illustrations of different types of motor endplates in rabbit EOMs. Single en plaque motor endplates (**A**–**C**, *arrowheads*), multiple en grappe motor endplates (**D**–**F**, *arrowheads*), multiterminal en plaque motor endplates (**G**–**J**, *arrows*), and sporadic complex en grappe motor endplates (**K**–**N**, *arrows*) were labeled with α-BTx (*red* in **B**, **E**, **H**, and **L**) and antibodies against neurofilament and synaptophysin (NF + SYN; green in **A**, **D**, **G**, and **K**). The contour of myofibers was outlined by immunolabeling with the antibody against laminin (*white* in **I** and **M**). Merged images in **C**, **F**, **J**, and **N**. *Scale bars*: 50 µm.

Approximately 76% ± 4.1% of the 366 myofibers studied exhibited single en plaque motor endplates, both in the GL and OL (Figs. 5A–C), whereas 2.8% ± 1.2% of the myofibers in both layers contained typical multiple small en grappe motor endplates (Figs. 5D–F). Single en plaque motor endplates were primarily concentrated at the mid-belly of the EOM, forming an endplate zone, as previously described.^19^

Additionally, multiterminal en plaque motor endplates were identified in approximately 21.7% ± 2.9% of myofibers of both GL and OL (Figs. 5G–J). The EOM myofiber segments containing the multiterminal en plaque motor endplates could be followed for up to 455 µm long and the distance between adjacent motor endplates varied between 16.4 and 146 µm. The length of each individual NMJ in the multiterminal en plaque motor endplates varied between 5.1 and 83.6 (27 ± 1.7) µm. In contrast to the typical multiple en grappe endplates aligned on only one side of the myofiber^20^ (Figs. 5D–F), small endplates lined up along both sides of a single myofiber were also sporadically found (Figs. 5K–N). We called them complex en grappe motor endplates to indicate that they were different from the classic multiple en grappe motor endplates. In one case, a single myofiber displayed 13 of these small complex en grappe motor endplates, with a distance between the first and the last endplate on the single myofiber studied extending to 334 µm. Unfortunately we were not able to determine with certainty whether these endplates were singly or multiply innervated.

### Mouse

**Figure 6.**
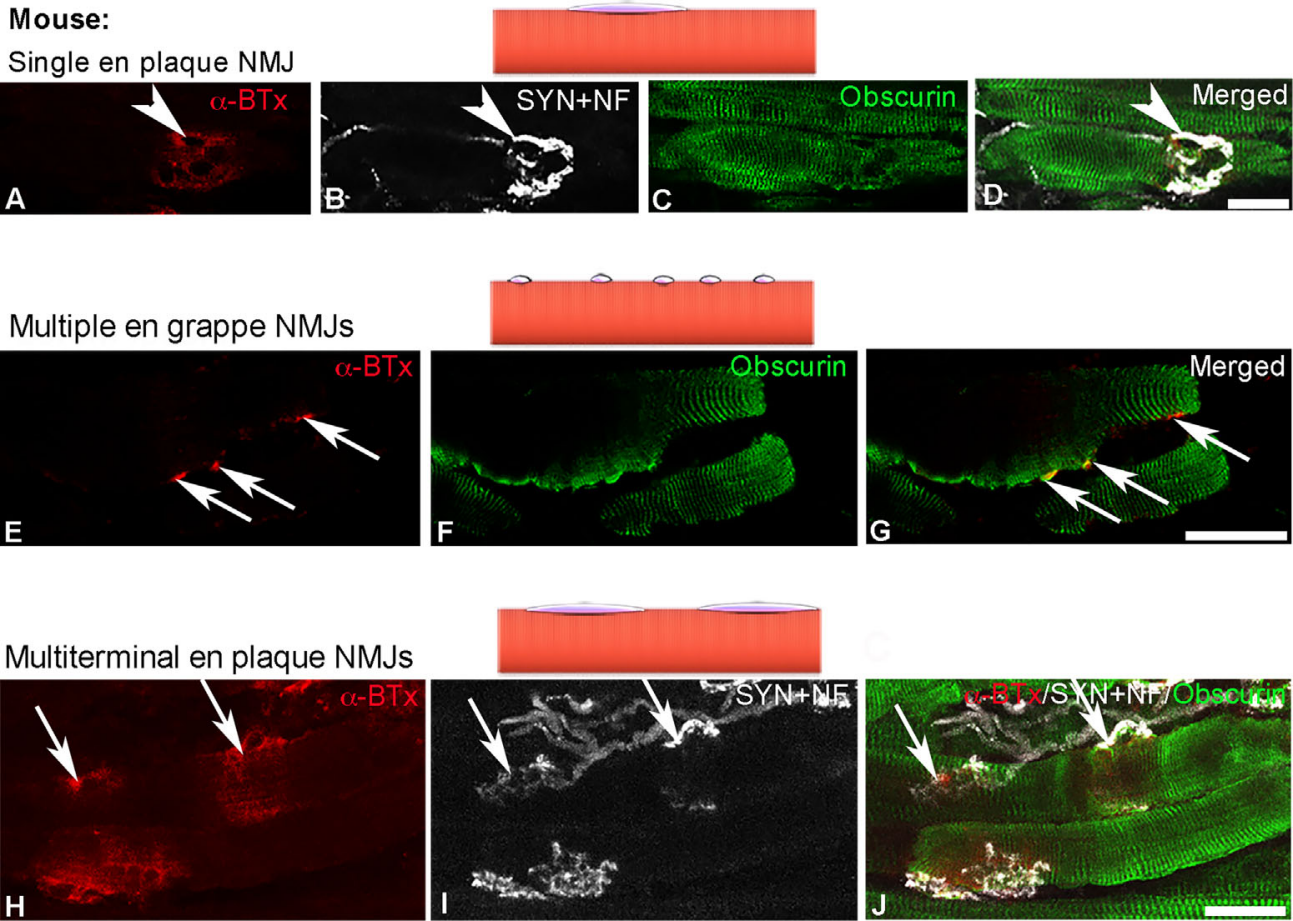
Confocal images and schematic illustrations of three types of motor endplates in mouse EOMs. Single en plaque motor endplates (**A**–**D**, *arrowheads*), multiple en grappe motor endplates (**E**–**G**, *arrows*), and multiterminal en plaque motor endplates (**H**–**J**, *arrows*) were labeled with α-BTx (*red* in **A**, **E**, and **H**) and antibodies against neurofilament and synaptophysin (NF + SYN; *white* in **B** and **I**), and myofibers were labeled with antibody against obscurin (*green* in **C** and **G**). Merged images showed in **D**, **G**, and **J**. *Scale bars*: 50 µm in **D** and **G**, 25 µm in **J**.

A total of 196 myofibers containing motor endplates were encountered in longitudinal sections, and approximately 88.5% ± 5.1% of them displayed single en plaque motor endplates (Figs. 6A–D), suggesting the predominance of this endplate type in mouse EOMs. These single en plaque motor endplates were generally large, compact and lobulated. These motor endplates were encountered in both the GL and the OL, and were predominantly concentrated around the middle belly region of the EOMs, where they formed a motor endplate band, consistent with previous reports.^21^ The typical small multiple en grappe motor endplates, running along one side of a single myofiber, were observed in a small percentage (1.4% ± 0.5%) of the myofibers examined in both GL and OL (Figs. 6E–G). The distribution and morphological patterns of these two types of motor endplate generally closely resembled those observed in rabbit EOMs.

Multiterminal en plaque motor endplates were observed in approximately 10.2% ± 4.8% of mouse EOM myofibers studied, in both GL and OL (Figs. 6H–J). The distance between adjacent NMJs in the same myofiber varied between 18.4 and 218.1 µm, and the length of each endplate varied between 5.6 and 58.1 (32.3 ± 4.2) µm.

**Figure 7.**
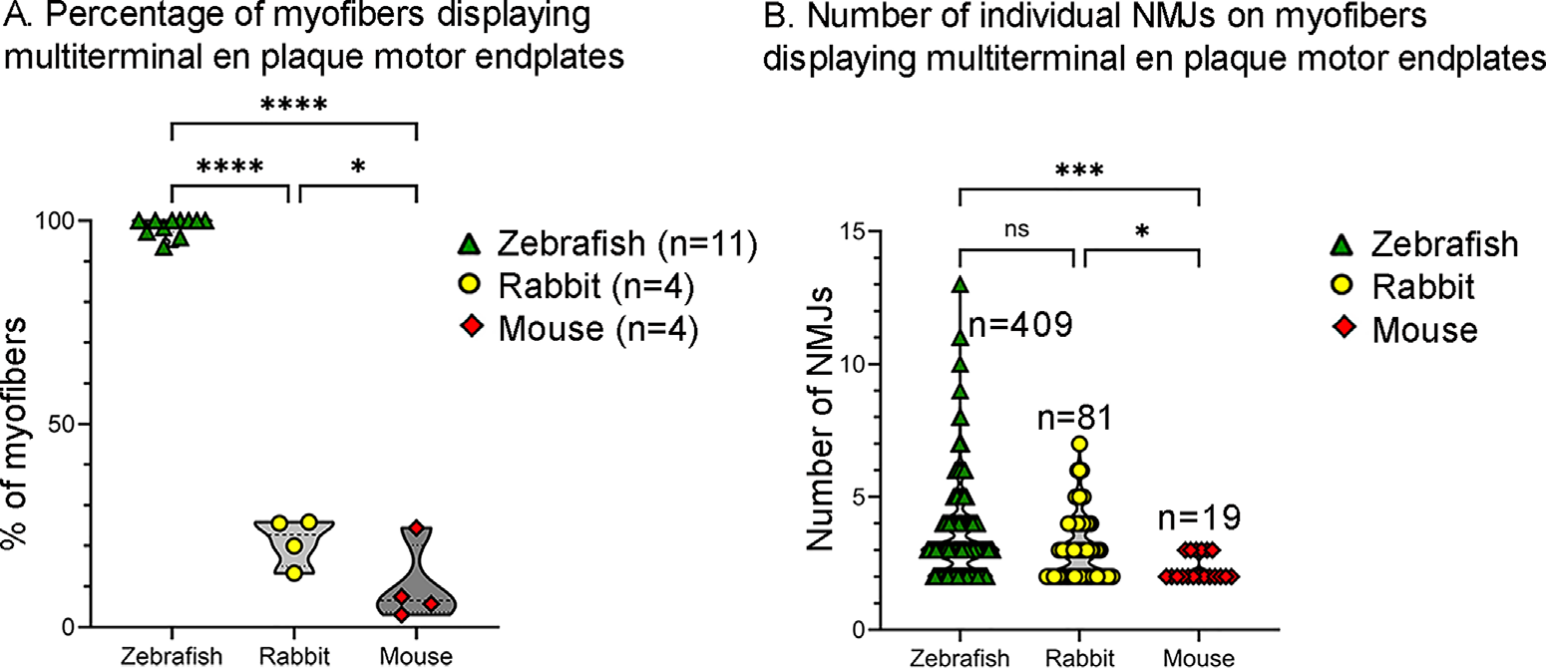
Quantification of myofibers containing multiterminal en plaque motor endplates in zebrafish, rabbits and mice. (**A**) Percentage of myofibers displaying multiterminal en plaque motor endplates. The number of animals studied in each group is given in parentheses. (**B**). The number of individual NMJs on myofibers displaying multiterminal en plaque motor endplates, with n representing the number of myofibers examined. *P ≤ 0.05; **P ≤ 0.01; ****P ≤ 0.0001; ns: not significant.

The proportion of the myofibers containing multiterminal en plaque myofibers was statistically significantly higher in zebrafish than in mouse (*P* ≤ 0.0001) and rabbit EOMs (*P* ≤ 0.0001), and the proportion of myofibers containing multiterminal en plaque motor endplates was significantly higher in rabbit than in mice (*P* = 0.02; Fig. 7A). In addition, the number of NMJs on myofibers displaying multiterminal en plaque motor endplates was significantly higher in zebrafish (3.4 ± 0.07) compared to mice (2.3 ± 0.1; *P* = 0.0004) and rabbits (3.0 ± 0.1; *P* = 0.03), and no significant difference was found between rabbits and mice (*P* = 0.06; Figs. 7B).

## Discussion

Three major types of motor endplates could be distinguished in the EOMs of all three species examined in the present study: zebrafish, rabbit, and mouse. These included the following: (A) typical large single en plaque motor endplates, (B) typical small multiple en grappe motor endplates, both of which have been described, previously,^6^^,^^7^ and (C) a third type characterized by the presence of several en plaque motor endplates along a single myofiber, referred to as multiterminal en plaque motor endplates. This third type of motor endplates has been thoroughly described in the human EOMs^9^ and briefly mentioned in zebrafish EOMs.^14^ Here, we present evidence that these multiterminal en plaque motor endplates also are present in the EOMs of mouse and rabbit, which shows that this is a conserved vertebrate EOM feature.

As mentioned in Results, the examination of motor endplates was mostly carried out in the central part of the EOMs and we were unable to follow the full length of a single myofiber because of the unavailability of the whole muscle sample from mice and rabbits. This methodological limitation most likely led to an underestimation of the proportion of myofibers with small multiple en grappe motor neurons in mice, and, to a less degree, in rabbits. Even though we clearly identified the three different innervation patterns in all examined animals, a relation between myofiber type and motor endplate type was not possible to establish because of the large number of antibodies needed and their species specificities.

The two typical types of motor endplates in EOMs, single en plaque and multiple en grappe endplates, have been extensively studied in mouse and rabbit EOMs, including their association with specific myofiber types.^2^^–^^4^^,^^6^ NMJ innervation in zebrafish EOMs has been rarely explored,^14^^,^^22^ therefore a more comprehensive investigation of the EOMs in zebrafish was carried out because it is necessary to better understand their unique characteristics. The current study is, to the best of our knowledge, the first to thoroughly examine the distribution of NMJs on whole-mount EOMs of zebrafish. Zebrafish EOMs exhibited a higher total number of NMJs in the whole muscle, a greater frequency of myofibers displaying multiterminal en plaque motor endplates, and a higher number of NMJs on individual myofibers compared to rabbit and mouse. These results align with previous observations on longitudinal sections of zebrafish EOMs, where NMJs were found densely distributed along the entire myofiber length.^22^ Nevertheless, the novel finding of multiterminal motor endplates was only possible in whole-mount zebrafish EOMs. This could not be revealed in longitudinal sections where each individual myofiber could not be followed with certainty for a longer distance. In mammalian vertebrates, multiple innervation of trunk and limb muscle fibers is seen only during early development, and it is absent in myofibers of adult limb and trunk muscle.^23^ In contrast, multitple innervation is common in non-mammalian vertebrates, including amphibians and fish. A number of studies investigating development and structure of NMJs in trunk muscles of fish, including teleosts,^24^^,^^25^ report that both slow and fast myofibers are multiply innervated. In other words, the high proportion of myofibers displaying multiterminal en plaque motor endplates in the EOMs of zebrafish is likely in agreement with their trunk myofibers beeing multiply innervated.

The present findings reveal a significantly higher number of myofibers displaying multiterminal en plaque motor endplates, adding a new layer of complexity to our understanding of zebrafish EOMs innervation and stand in contrast to the traditional patterns observed in mammals.^6^^,^^7^^,^^9^ Notably, a significant difference in the number of motor endplates was found between the large OL myofibers and the slow myofibers in zebrafish EOMs.

We found differences in the morphology of multiple en plaque motor endings in zebrafish including the form of the NMJs, which varied from pretzel-shaped, linear, jagged, and in some cases with clear ramification to adjacent myofibers, as compared to those of mammals. It is difficult to speculate whether these morphological differences among NMJs will have implications on their physiological properties. The extraordinary number of multiterminal en plaque motor endplates in zebrafish EOMs may play a crucial role in their high-frequency transmission capabilities, which lead to fast contraction of EOMs under special circumstances such as predation and escape responses in their aquatic environment (i.e., evolutionary adaptation). Many studies have focused on comparative morphology of NMJs in skeletal muscles across species, revealing species-specific differences in size, shape, distribution, and structure.^26^^,^^27^ For example, in frogs, NMJs are relatively large, but their postsynaptic folding is less complex.^26^ In contrast, humans have smaller NMJs but more extensive postsynaptic folding, suggesting that NMJs have undergone evolutionary modifications to optimize their function across different species.

The innervation of EOMs shared many similarities across species, likely reflecting their shared functional demands. In both mammals and zebrafish, the EOMs are more densely innervated than other skeletal muscles and exhibit a higher ratio of motor endplates to myofibers.^28^ Consequently, the motor units in EOMs are considerably smaller, typically consisting of only 10 to 15 myofibers per unit, whereas motor units in other skeletal muscles may contain hundreds of myofibers.^28^ This dense innervation is thought to support the precise and rapid eye movements required for visual tracking and stability. Consistent with this, transcriptomics data from zebrafish reveal significantly higher expression of genes encoding AChR subunits in EOMs compared to trunk muscles, generally mimicking immunohistochemistry data from human EOMs with both neonatal and adult subunits present in the EOMs. This suggests an enhanced cholinergic signaling capacity in EOMs, which may further contribute to their specialized neuromuscular properties. These findings align with previous studies highlighting the unique structural and functional characteristics of EOMs and provide molecular evidence supporting their distinct innervation patterns.^4^ In the present study it was not possible to morphologically study the distribution of the AChR subunits because of the lack of specific antibodies that work on zebrafish muscle.

Pioneering studies of innervation patterns in EOMs using electrophysiological recordings/techniques and histochemical staining revealed two primary types: singly (SIF) and multiply (MIF) innervated myofibers.^6^^,^^20^^,^^29^ This classification was useful because it could easily be related to the unique functional demands of EOMs (i.e., SIF enable rapid and fine control of eye movements whereas MIF provide stable, sustained muscle contraction necessary for maintenance of eye position).^30^ Subsequent studies have confirmed the general existence of this pattern, and the SIF and MIF pattern of EOM innervation has been widely used.^7^ Although the presence of two en plaque motor endplates on a single myofiber in human EOM was reported,^6^ this was initially considered as an “exceptional” finding because of its rarity,^6^ and the presence of several en plaque motor endplates on a single myofiber has not been recognized until recently.^9^^,^^13^^,^^14^ In a comprehensive study of human EOMs, we found that multiterminal en plaque motor endplates are actually present in a substantial proportion of EOM myofibers.^9^ This is in agreement with electrophysiological studies reporting that the sum of twich and tetanic tensions in response to individual nerve branch stimulation is greater than that when the whole nerve to the EOM is simultaneously stimulated.^3^^,^^31^ In the present study, it was not possible to determine whether the multiple innervated myofibers received one or more different axons.

The present study confirms that multiterminal en plaque motor endplates are present in EOMs of other species (zebrafish, rabbit, and mouse). This strongly suggests that multiterminal en plaque motor endplates represent a fundamental and common characteristic of EOMs across vertebrates and are an important evolutionary conserved trait. However, the precise function of these endplates as opposed to single en plaque and multiple en grappe endplates remains to be defined. In summary, multiterminal en plaque motor endplates are present in human, zebrafish, rabbit, and mouse EOMs, suggesting that they are conserved feature of the EOMs across vertebrates. Further physiological studies should take this into consideration.

## Supplementary Material

Supplement 1
