# Supplementary material for: Multiterminal En Plaque Motor Endplates in Extraocular Muscles Are Conserved Across Vertebrate Species
Source: Invest Ophthalmol Vis Sci. 2025 Apr 28;66(4):77. doi: 10.1167/iovs.66.4.77 (PMC12060065; doi:10.1167/iovs.66.4.77)
Supplement: Supplement 1 [file iovs-66-4-77_s001.pdf]

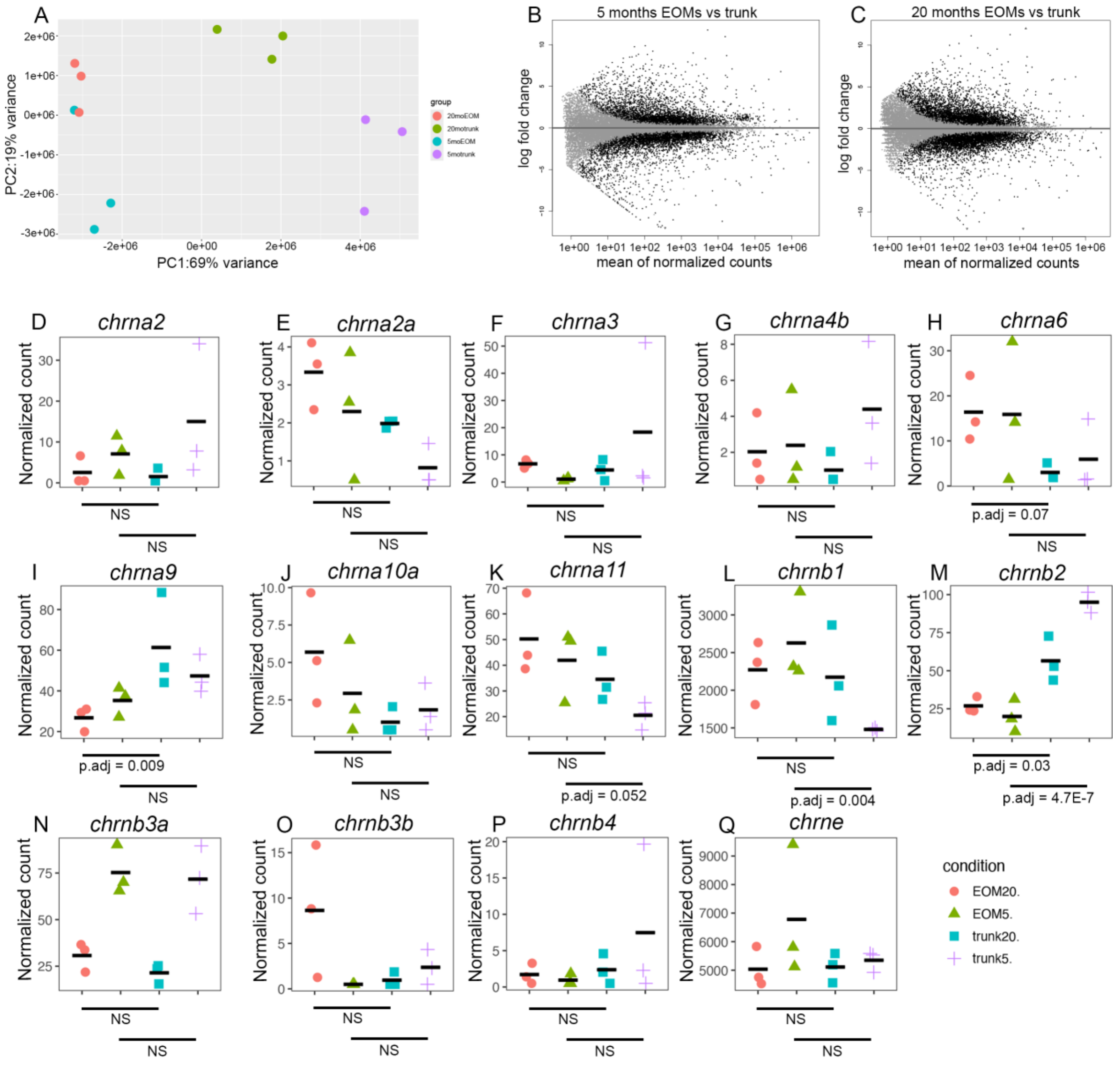

Supplementary figure 1. Clustering and comparison of AchR related genes in EOMs and trunk muscle tisuee A) PCA plot of the included samples displaying the two largest points of variance for the top 500 genes (PCA1: tissue, PCA2: age). B-C) MA-plots of the included samples comparing 5-month-old EOMs to trunk muscle (B, 5286 differentially expressed genes) and 20-month-old EOMs to trunk muscle (C, 6557 differentially expressed genes), an adjusted P-value of 0.01 was considered significant. D) Normalized gene expression for all AChR-genes not consistently higher in EOMs compared to trunk muscle tissue.

Supplementary Table S1

Primary antibody	Host	Isotype	Dilution	Code number
Synaptophysin	Mouse	IgG1	1:50	SY38
Neurofilament	Mouse	IgG1	1:800	M0762
Synaptic vesicle	Mouse	IgG1	1:100	SV2
Acetyl-alpha tubulin	Mouse	IgG1	1:100	T7451
Obscurin	Rabbit	IgG1	1:100	IQ
Laminin	Chicken	IgG1	1:800	LS-C96142
S58	Mouse	IgA	1:10	4/28/16
**Secondary antibody /ligand**	**Against**		**Dilution**	**Product nr**
Alexa Fluor 488	Donkey anti-mouse	IgG	1:300	715-545-151
Alexa Fluor 488	Donkey anti-rabbit	IgG	1:400	711-545-152
Alexa Fluor 647	Donkey anti-mouse	IgG	1:400	715-605-150
Alexa Fluor 647	Donkey anti-chicken	IgG	1:400	703-605-155
Rhodamine Red-X	Donkey anti-mouse	IgG	1:500	715-295-151
Phalloidin	Alexa Fluor 488	-	1:100	A12379
α-Bungarotoxin,	Alexa Fluor 647	-	1:300	B35450
α-Bungarotoxin	Tetramethylrhodamine	-	1:300	T1175
